# Extraction and characterization of pectin from coffee waste and the effects on pectin-maize starch gel

**DOI:** 10.1016/j.fochx.2026.103804

**Published:** 2026-03-30

**Authors:** Wei Zhang, Chunyan Zhang, Jiahe Dai, Danxia Shi, Fang Yang, Hong Li, Xiaohui Liu

**Affiliations:** a College of Food Science and Technology*,* Yunnan Agriculture University*,* Kunming 650500*,* People's Republic of China; b Yunnan College of Modern Coffee Industry, Kunming 650500, People's Republic of China; c College of Tea Science*,* Yunnan Agricultural University*,* Kunming 650500*,* People's Republic of China; d Processing Research Institute, Yunnan Academy of Agricultural Sciences, Kunming 650201,People's Republic of China

**Keywords:** Coffee pectin, Maize starch, Degree of esterification, RG-I, Composite gels

## Abstract

The pectin from coffee waste water and coffee pulp by different extraction methods were obtained and the interaction between pectin and maize starch (MS) were investigated in the present study. Degrees of methyl-esterification (DM) for pectin with acid (AEP), chelating agent (CSP) and water soluble pectin (WSP) were 45.8%, 22.6% and 28.6%, respectively. The results of pasting properties showed that pectin from coffee waste (CW) reduced the viscosity of composite gels, and ability of the pectin to maintain the viscoelasticity of composite gels followed the order AEP, CSP and WSP. The AEP, CSP and WSP significantly decreased the relative crystallinity from 4.03% to 2.91%, 1.37%, 3.10% and enhanced the short-range order. These results suggested that both methylation and RG-I are likely to interact with MS chains and the interaction mainly depended on hydrogen bonds without changing the B-type of crystallinity in the composite gels.

## 1 Introduction

Sustainable production and consumption are needed to address the increasing environment-related issues, especially for biomass resources. Burning or disposal as organic material may lead to pollutant in soil and greenhouse gases. For economic and environment reasons, biomass resources from agro-industrial waste have attracted great interest as cell wall polysaccharides. CW is harmful to the environment with the traditional model. In pursuit of more refined coffee beans and better control of fermentation, wet processing is usually adopted to removal of the mucilage layer from the seed, which characterized by simple sugars, arabinogalactans, and pectins (Tsigkou et al., 2025). Thus; every ton of coffee cherry generate 0.5 ton of coffee pulp and 8 to 20 m^3^ of residual water; and nearly 10.5 million tons of coffee beans were produced in 2020; large amounts of coffee wastewater are produced in the processing site (Reichembach, Guerrero, et al., 2024). Coffee pulp; another CW; which was mainly composed of exocarp and mesocarp of coffee cherries; also contain abundant carbohydrates (21–50%); become competitive pectin original materials (Reichembach, Kaminski, et al., 2024).

Pectin is a non-starch polysaccharide with α-1,4 linked D-galacturonic acid as backbone, partially methylated at C-6 and acetylation at O-2 and/or O-3 from plant cell wall. As a gelling, thickening, stabilizing and emulsifying agent in the food industry, pectin is of excellent safety with no specified limit on daily intake. The pectin isolated from coffee pulp with HNO_3_ was reported to present commercial features and could form gel with sucrose and xylitol (Reichembach & de Oliveira Petkowicz, 2020). Also; the coffee pulp pectic fractions were isolated by sequential exactions to be low-methoxylated with arabinogalactan-proteins (AGPs) (Reichembach, Kaminski, et al., 2024). However, the sequential extraction of pectin usually lead to low yield and is difficult to apply in industry.

As shown in previous studies, pectin has the ability to improve the swelling power, foaming behavior, viscoelasticity, crystallinity and digestibility of starch (Chen et al., 2023; Zhang et al., 2025). Li et al. (2020) have showed that the slowly digestible starch (SDS) and resistant starch (RS) in potato starch were increased due to the hiding of binding sites of amylase by pectin. Chen et al. (2025) suggested that the water-soluble pectin; chelating agent soluble pectin and sodium carbonate soluble pectin could inhibit starch digestion with different principles and less esterified pectin showed better effect. Chen et al. (2022) revealed that pectin could penetrate into the interior of potato and pea starch through the surface pores and channels, while for rice starch, the pectin accommodated on the surface, the viscosity profile of starch samples all changed by reducing granule swelling. The previous studies have found that the influence of pectin on starch properties is closely related to the structure of pectin.

Maize starch is a multifunctional ingredient in cooked and baked products, and many defects has been found in the application, such as low viscosity, heating instability, high syneresis, and easy retrogradation. The improvement of the functional characteristics of maize starch has become an interesting topic. Studies have found the pectin with different DE values could inhibit starch retrogradation, enhance the texture of starch gel and alter the pasting properties (Dorantes-Campuzano et al., 2024). However, the role of pectin structure characters (e.g. RG-I) and its synergistic effects on the properties of pectin-maize starch gels have not been researched.

As the interaction between pectin and maize starch is closed to the structure of pectin, it is hypothesized that pectin originated from various coffee waste and obtained by different extraction method, can result in the structural differences, provide functional group for interaction and regulate the properties of pectin-maize starch gels for more extensive applications. In this study, the pectin from coffee waste water and coffee pulp were extracted characterized, including Fourier transform infrared (FT-IR) spectroscopy, nuclear magnetic resonance (NMR) spectra, and Scanning electron microscope (SEM). In order to better understand the interaction mechanism between different molecular structure pectin and maize starch, pectin from CW on the physicochemical properties of maize starch-pectin gel by measuring the thermal and dynamic rheogogical properties. The results of study will provide a better understanding of the character of coffee pectin-maize starch interactions and be of significant importance in expanding the applications of pectin from CW in the starchy food industry.

## 2 Materials and methods

### 2.1 Materials and reagents

Maize starch was purchased from Macklin Inc. (Shanghai, China). CW (*Coffea arabica* L.) was kindly provided by Baoshan Hooiock coffee villa (Baoshan, Yunnan, China). Galacturonic acid was purchased from Sigma-Aldrich (Shanghai, China). All other chemicals and reagents were analytical grade. All solutions were prepared using distilled water.

### 2.2 Pectin extraction

Coffee pulp (150 g) was first freeze-dried and grinded, then mixed with 80% (*v*/v) boiling ethanol (1 L) for 20 min reflux. The alcohol insoluble residue (AIR) was generated by filtrating the ethonal solution and washing 3 times with 100 mL of absolute ethyl alcohol. The AIR was recovered from the ethanol solution by vacuum filtration and subsequently dryed at 35 °C at oven (DHG-9240 A, Shanghai Jing Hong Laboratory Instrument Co.,Ltd. Shanghai, China). Then, the AIR was milled and stored at -20 °C for further extraction (Reichembach et al., 2024).

The acid and chelating soluble pectin from coffee pulp were obtained with the method described by Reichembach and de Oliveira Petkowicz (2020) and Reichembach, Kaminski, et al. (2024). The AIR was mixed with boiling 0.1 M HNO_3_ for 30 min and 0.5% ammonium oxalate at 70 °C for 2 h at the ratio of 1:15 (*w*/*v*), respectively. The extract was filtered with a polyester fabric and centrifuged at 5000 rpm for 20 min, then precipitated with 2 volumes of absolute ethanol for 16 h at 4 °C, giving rise to pectin from coffee pulp with acid (CP-AEP) and chelating agent (CP-CSP) extraction.

The water soluble pectin from coffee waste water (CWW-WSP) was obtained by vacuum filtration (10 μm Qualitative filter paper, 0.08 MPa), and precipitated with 2 volumes of absolute ethanol for 16 h at 4 °C.

As for purification of CP-AEP, CP-CSP and CWW-WSP, the crude polysaccharide obtained after freeze-drying was dissolved in pure water and subjected to decolorization with AB-8 macroporous resin. The solution was then dialyzed (3000 Da) against pure water for 24 to 48 h, concentrated, freeze-dried and stored in a refrigerator at 4 °C for further use.

### 2.3 Chemical characterization of the pectin

#### 2.3.1 Monosaccharide analysis

The monosaccharide composition of pectin was performed according to previous method (Zhu et al., 2021) with modifications. Pectin samples (5 mg) was hydrolyzed using 1 mL TFA (2 M) at 121 °C for 2 h in a sealed tube, dried with nitrogen. Then washed by methanol and blown dry, and repeated the process for 3 times. The residue was re-dissolved in deionized water and filtered through microporous filtering film (0.22 μm) for measurement. The samples were analyzed by high-performance anion-exchange chromatography with pulsed amperometric detection (HPAEC-PAD) consisted of Dionex ICS 5000+ system (Thermo Fisher Scientific) on a CarboPac PA-20 anion-exchange column (150 mm × 3 mm, 6.5 μm). The injection volume was 5 μL, and the flow rate were 0.5 mL/min at 25 °C.

#### 2.3.2 DM

Samples (1.0 mg) were mixed with KBr powder and pressed into pellets for Fourier transform infrared (FT-IR) measurement (Bruker, Rheinsetten,Germany). The spectroscopy was conducted in the range of 4000–400 cm^−1^ with a resolution of 4 cm^−1^ for a total of 32 scans by Fourier Transform Infrared Spectrometer Nicolet iZ-10 (Thermo Fisher Scientific, Inc., Waltham, MA, USA). The acquired spectral data were subsequently analyzed using PeakFit software. Peaks assigned to the methyl-esterified (1749 cm^−1^) and the non-esterified carboxyl groups (1630 cm^−1^) were used to calculate the DM values (Reichembach, et al., 2020).

#### 2.3.3 Protein content

Protein content of pectin samples were determined by Bradford method with bovine serum albumin (BSA) as standard and measuring absorbance at 595 nm (Bradford, 1976). Each experiment were performed in triplicate.

#### 2.3.4 Moisture contents of pectin

The moisture contents were estimated following the standard method of AOAC (Chemists, 2000). Pectin (0.5 g) was placed into a tared crucible and dried in an oven at 100 ± 5 °C until constant weight and cooled in a desiccator. The moisture contents were calculated as ratios of constant weight and initial weight. Each experiment were performed in triplicate.

### 2.4 Preparation of composite gels

The experimental procedure was conducted following the methodology described by Yin et al. (2021) and based on preliminary experiments. Firstly, MS was blended with 3% (*w*/w) of pectin derived from different sources to formulate mixed powders. Aqueous suspensions (10%, w/w) were prepared by dispersing the mixed powders in distilled water. The suspensions were subjected to gelatinization by heating in a water bath maintained at 95 °C for 30 min with constant agitation. Subsequently, the gelatinized systems were cooled to room temperature to form composite gels, the resulting composite gels were lyophilized, grinding pulverized and pass mesh sieve to obtain homogeneous powders. A control sample without pectin incorporation and consisting solely of maize starch was prepared under identical conditions. The gel samples were labeled as MS-AEP, MS-CSP, MS-WSP, respectively.

### 2.5 Pasting characteristics of mixed powder

The pasting properties were analyzed using a Rapid Visco Analyzer (RVA Super 4, Newport Scientific Pty Ltd., Warriewood, Australia). A mixture powder of pectin and maize starch was taken and distilled water was added at a ratio of 3 g/25 g to form a mixed solution. A controlled heating and cooling cycle was carried out under constant shear (160 rpm), maintained at 50 °C for 1 min, then heated from 50 °C to 95 °C at a rate of 12 °C/min and remained at 95 °C for 2.5 min, cooled at a rate of 12 °C/min to 50 °C, and remained at 50 °C for 2 min to obtain viscosity parameters according to the the (AACC, 2010) International Method 76–21.

### 2.6 Dynamic rheological properties of composite gels

The rheological properties of composite gels were performed according to the method by described by Jinfeng, Yanli, Wanlu, Yunxiang, & Shenggui, (2023) using a Discovery HR-1 Hybrid Rheometer (TA Instruments, New Castle, DE, USA) with modifications. The prepared mixed powder was hydrated with distilled water at a solid-to-liquid ratio of 5 g/100 g to form are homogeneous gels suspension was heat the mixed gels at 95 °C for 30 min, then put into measuring system of rheometer, allowed to relax and cool it down to the measurement temperature. The storage modulus (G′), loss modulus (G′′) and loss tangent (tanδ) were recorded at a parallel plate geometry (40 mm cone diameter, 1.0 mm gap size). The frequency scanning was performed at range of 0.01–100 rad/s-1 and strain at 1%, which was with the linear viscoelastic range (LVR) of the composite gels.

### 2.7 FT-IR spectroscopy of composite gels

FT-IR spectroscopy of composite gels were performed according to the reported method (Chen et al., 2023). In brief, the composite gels was mixed with KBr (1:100), pressed into tablets. Full-band scanning by 64 scans in the range of 4000–400 cm^−1^ at a resolution of 4 cm^−1^ was carried out. The hydrogen bonding energy and distance was calculated based on the second-derivative spectra of infrared spectrum using the Savitsky-Golay method with the OMNIC software according to a reported method (Lu et al., 2021).

### 2.8 Morphological observation

The microstructure of samples was characterized using field emission scanning electron microscopy (FE-SEM; Zeiss Merlin Compact, Carl Zeiss AG, Germany). Sample preparation followed the method described by Sandhu et al. (2021). 500 μL of sample suspensions dissolved in ethanol, dropped onto the conductive glue on the copper plate, spread it evenly as much as possible, and leave it at 37 °C for 12 h. After gold coating using a Cresciton 108 Auto ion sputter coater (Cresciton Scientific Instruments Ltd., UK) morphological observation was conducted at an accelerating voltage of 15 kV.

### 2.9 Solid state 13C cross polarization magic angle spinning (13C CP/ MAS) spectra of composite gels

300 mg of the composite gels were placed in a 2 mL centrifuge tube and put in a saturated sodium chloride solution environment and allow the moisture to balance for one week. According to the method proposed by Ma et al. (2023). Solid-state NMR spectra of freeze-dried composite gels were acquired using the Bruker AVANCE III 400WB spectrometer (Bruker BioSpin GmbH, Rheinstetten, Germany). The resonance frequency of ^13^C was 100.6 MHz, the rotor rotation speed was 6 KHz, the contact time was 1.2 ms, the delay time was 2.0 s, and the accumulation times were 1000–1600 times, the original data were exported using MestReNova software, and then quantitative analysis was conducted on them by using PeakFit v4 software.

### 2.10 X-ray diffraction (XRD)

The milled gel samples were equilibrated to 20 ± 0.5% moisture content using a saturated humidity chamber at 15 °C for 72 h. XRD analysis was performed by using the PANalytical X'Pert Pro diffractometer (Malvern Panalytical, Almero, The Netherlands) according to the previous method with some modifications (Bao et al., 2018). The instrument were operated at 40 kV and 30 mA and used Cu Kα radiation (wavelength 0.15406), measured by a NaI crystal scintillation counter to determine the X-ray intensity, the scanning range of the diffraction angle 2θ is from 4 to 60 degrees, with a step size of 0.02 and a scanning rate of 4°/min. The DS-SS-RS setting were 0.5 mm–0.25 mm-0.1 mm, respectively. Data analysis was performed using MDI Jade 9.0 software.

### 2.11 Statistical analysis

All experiments were repeated in triplicate and results were expressed *a*s mean ± standard deviations. Data was analyzed using one-way analysis of variance (ANOVA) and means were compared by Tukey's test using SPSS 25.0 (*p* < 0.05).

## 3 Results and discussion

### 3.1 Extraction and chemical characterization of pectins from CW

The chemical composition of pectic extractions (CP-AEP, CP-CSP and CWW-WSP) were determined and exhibited in Table 1.The yields of CP-AEP (8.4%) and CP-CSP (9.3%) were lower than which of pectin (14.6%) extracted by acid from coffee pulp AIR and the sum of pectic fractions (15.8%) from coffee pulp (Reichembach, Kaminski, Maurer and de Oliveira Petkowicz, 2024, Reichembach and de Oliveira Petkowicz, 2020). Similar amounts of pectin (11.37%, based on the dry pulp) were reported in previous study (Bonilla-Hermosa et al., 2014). This might related to the varieties and cultivation condition of coffee. Also; the yields were much lower than pectin from apple peel (16.68%) and citrus peels (21.95%) which is the main commercial pectin origin (Wang et al., 2014). The yield of CWW-WSP was much lower (0.97%), this may be close related with the amount of water used to wash the coffee beans. The results suggested that in order to increase resource utilization rate, the CWW should undergo concentration treatment before pectin production.

**Table 1:** Yield, chemical composition and molecular features of CW.

Samples	CP-AEP	CP-CSP	CWW-WSP
Yield (%)⁎	8.4 ± 0.2^b^	9.3 ± 0.2^a^	0.97 ± 0.04^c^
Protein (g/100 g)	0.4 ± 0.02^c^	2.7 ± 0.08^b^	3.10 ± 0.22^a^
Moisture (%)	9.56 ± 0.72^a^	9.64 ± 0.80^a^	8.92 ± 0.69^b^
Monosaccharide (relative %)			
Rha	8.87 ± 0.31^b^	7.00 ± 0.14^c^	9.08 ± 0.54^a^
Fuc	0.19 ± 0.00^c^	0.42 ± 0.12^a^	0.34 ± 0.08^b^
Ara	4.62 ± 0.51^c^	19.37 ± 0.84^b^	25.22 ± 1.22^a^
Xyl	2.97 ± 0.15^b^	2.32 ± 0.15c	4.63 ± 0.21a
Man	0.91 ± 0.00^c^	1.05 ± 0.00^a^	0.97 ± 0.00^bc^
Gal	15.64 ± 1.42^b^	13.16 ± 0.98^c^	16.91 ± 1.23^a^
Glc	8.15 ± 0.74^b^	6.08 ± 0.55^c^	9.24 ± 0.75^a^
GalA	55.89 ± 2.60^a^	48.18 ± 1.64^b^	30.69 ± 1.12^c^
HG⁎⁎	47.02 ± 1.45^a^	41.18 ± 1.79^b^	21.61 ± 0.94^c^
RG-I⁎⁎	38.00 ± 0.78^c^	46.53 ± 1.26^b^	60.29 ± 1.49^a^
Rha/GalA	0.16 ± 0.02^b^	0.15 ± 0.00^b^	0.30 ± 0.01^a^
(Ara + Gal)/Rha	2.28 ± 0.15^b^	4.65 ± 0.33^a^	4.64 ± 0.24^a^
DM⁎⁎⁎	45.8 ± 0.25^a^	22.6 ± 0.17^c^	28.6 ± 0.32^b^

⁎ Based on the AIR.

⁎⁎ HG = GalA-Rha and RG-*I* = 2(Rha) + Ara + Gal.

⁎⁎⁎ DM was determined by FT-IR.

As shown in Table 1, the monosaccharide compositions of the CP-AEP, CP-CSP and CWW-WSP were different. The GalA content of pectins varied from 30.69% to 55.89%, were all lower than 65% and did not satisfy the commercial pectin requirements. Arabinose was the main neutral monosaccharide of CP-CSP and CWW-WSP but not for the CP-AEP, in which arabinose might release by hydrolysis (Reichembach et al., 2024).

The HG and RG-I of pectin were also calculated by the monosaccharide composition (Table 1). The HG content of CP-AEP, CP-CSP and CWW-WSP were 47.02%, 41.18% and 21.61%, respectively. The CWW-WSP contained more RG-I than HG, which manifested the structural selectivity of different extraction methods for pectin: acid extraction was more likely to obtain pectin dominated by linear HG, while water-soluble extraction for CWW-WSP enriched pectin with highly branched RG-I structures. Ara and Gal were the main linkage monosaccharides of the side chains for pectin. The mole ratio of Rha/GalA of CP-AEP (0.16), CP-CSP (0.15) and CWW-WSP (0.30) were relatively high, reflecting the degree of branching. According to the structure of pectin, the rhamnose is the linkage of main chains (HG) and RG-I, the results indicated that the high proportions of RG-I in CP-AEP, CP-CSP and CWW-WSP. Also, the higher values of (Ara + Gal)/Rha in CP-CSP (4.65) and CWW-WSP (4.64) suggested that the length of side chains in CP-AEP was relatively shorter. The short RG-I for CP-AEP in coffee was also reported by Reichembach, et al., (2020), which might be due to the hard acid extraction conditions (boiling 0.1 M HNO_3_ for 30 min) resulting in degradation.

**Fig. 1:**
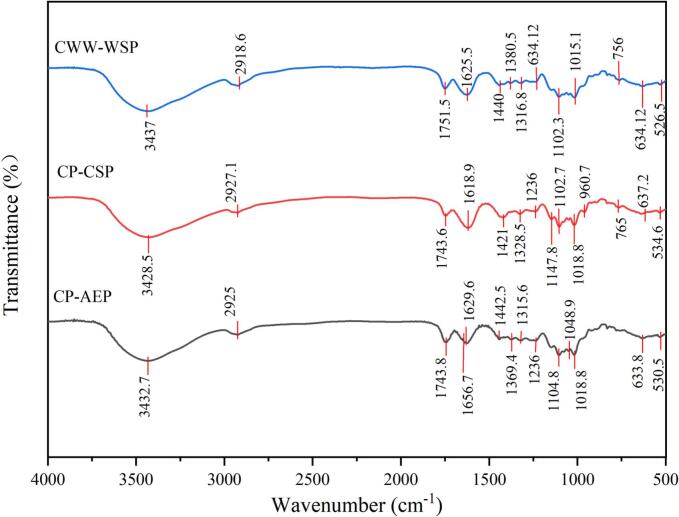
FT-IR spectra showing the methyl-esterified peak and unesterified carboxyl peak of CP-AEP, CP-CSP and CWW-WSP.

CP-AEP, CP-CSP and CWW-WSP were classified as low methoxyl (LM) pectin as reflecting by the peak areas ratio of methyl-esterified and unesterified carboxyl groups from FT-IR spectra (Fig.1), and consistent with the results reported by Reichembach, Kaminski, Maurer, & Petkowicz, (2024). These may attribute to the high solubility of LM pectins and the coffee pulp may also be subjected to the endogenous enzyme. However, the DM values of CP-CSP and CWW-WSP were much lower than CP-AEP, the results showed the selective binding with low-ester pectin of chelating solvents used for CP-CSP production, while result of CWW-WSP may be related with mucus layer and the role of endogenous enzymes before washing with water can not be ignored. However, HM pectin (63%) was also reported by acid extraction from coffee pulp (Reichembach and de Oliveira Petkowicz, 2020). This suggested that the acid extraction of pectin promoted the deesterification. The deesterification was promoted as pectin were subjected to different pH values (Yang et al., 2018). At this study, the HNO_3_ (0.1 M) was used to extracted the pectin and the time used was much longer, suggesting that the deesterification could occur and resulting the LM pectin.

The protein content of pectin obtained with acid (0.4%) was much lower than that with chelate (2.7%) and the pectin from coffee waste water (3.1%), which were close to hand dissected (3.4%) and commercial mucilages from coffee (4.9%) (Avallone et al., 2000).

### 3.2 Pasting properties

**Fig. 2:**
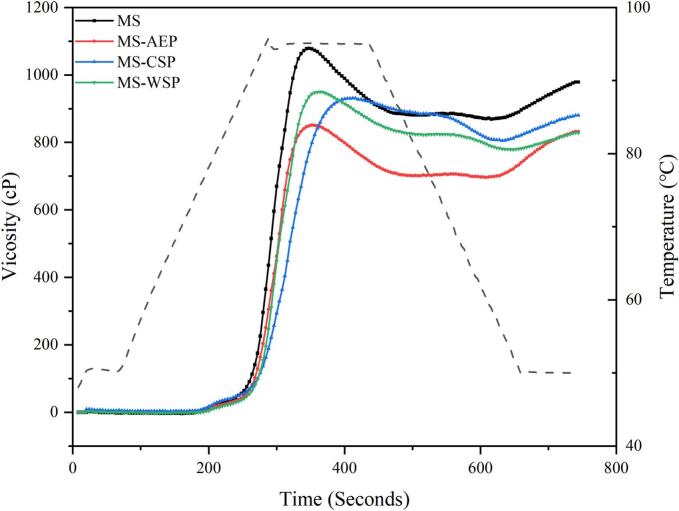
Pasting curves of starches with various pectin from CW.

The stability of the paste and short-term retrogradation tendency could be reflected by the RVA characteristics. The gelatinization curves and parameters recorded of MS and pectin-MS composite gels are shown in Fig.2 and Table 2. The addition of CP-AEP, CP-CSP and CWW-WSP decreased the overall viscosity of starch paste, suggesting that the coffee pectin could be competitive to starch for available water, and reduced the swelling of starch granules, PV reduction involves three synergistic mechanisms: (i) hydrophobic adsorption (dominant in high-DM CP-AEP), where methyl esters block water ingress (Chen et al., 2023); (ii) electrostatic repulsion/steric hindrance (CP-CSP), from low DM and RG-I side chains (Chen et al., 2023); and (iii) viscosity-mediated water retention (CWW-WSP), where highly branched RG-I increases aqueous phase viscosity (Liu et al., 2025; Yin et al., 2021). DM effects are threshold-dependent: substantial DM differences (CP-AEP vs. CP-CSP: 45.8% vs. 22.6%) produce clear PV differences (851 vs. 931 mPa·s). However, minor DM differences (CP-CSP vs. CWW-WSP: 22.6% vs. 28.6%) are overshadowed by RG-I architecture-CWW-WSP's higher RG-I (60.29%) and longer side chains ((Ara + Gal)/Rha = 4.64) enhance water-holding, explaining its marginally higher PV (950 mPa·s). The results were similar with pectin-corn starch gels, but opposite with the apple pectin-wheat starch gels, that might be due to the pectin-starch hydrogen bonding formation and close-packed structure of pectin-starch (Liu et al., 2025; Zhang et al., 2018).

**Table 2:** Gelatinization parameters of maize starch (MS) and maize starch-coffee waste pectin mixture.

Samples	PV (mPa·s)	TV (mPa·s)	BD (mPa·s)	FV (mPa·s)	SB (mPa·s)	PT (°C)
MS	1079 ± 22^a^	869 ± 19^a^	210 ± 7^a^	979 ± 14^a^	110 ± 6^b^	91.10 ± 0.21^b^
MS-AEP	851 ± 37^c^	696 ± 21^c^	155 ± 11^b^	832 ± 16^c^	136 ± 8^a^	91.85 ± 0.26^ab^
MS-CSP	931 ± 15^b^	805 ± 26^b^	126 ± 6^c^	880 ± 12^b^	75 ± 5^c^	92.65 ± 0.78^ab^
MS-WSP	950 ± 21^b^	779 ± 14^bc^	171 ± 14^ab^	829 ± 27^c^	50 ± 6^d^	94.25 ± 0.89^a^

Values are expressed as means ± standard deviations. Different letters represent a significant difference between data in the same column (*p* < 0.05).

The peak viscosity (PV) represents the maximum viscosity of starch during the gelatinization, which is related to the water absorption characteristics, swelling capacity and shear resistance. The viscosity reduction followed the order CP-AEP > CWW-WSP > CP-CSP, reflecting distinct structural mechanisms. CP-AEP (DM 45.8%) reduced viscosity primarily through hydrophobic adsorption of methyl ester groups onto starch granule surfaces, restricting water ingress. CP-CSP (DM 22.6%, RG-I 46.53%) exerted intermediate effects via electrostatic repulsion and steric hindrance from extensive RG-I side chains (Ara + Gal/Rha 4.65). CWW-WSP (RG-I 60.29%) exhibited viscosity-mediated water retention due to highly branched RG-I architecture, resulting in less pronounced viscosity reduction despite high RG-I content. One hand, CP-AEP, CP-CSP and CWW-WSP were all LM pectin, which is hydrophilic results in competitive with starch for water. The pectin molecules were more likely to expand and form hydrogen bond with water with weakening of electrostatic repulsion. Thus the maximum swelling of starch could not be reached resulting in decrease of the viscosity (Bao et al., 2018). On the other hand; the RG-I portion was prone to absorbed on the starch surfaces and inhibit amylose swelling; and the coating effects was close related with the chain length of side chains reflecting by (Ara + Gal)/Rha (Yang et al., 2025). Though the RG-I content of CWW-WSP was much higher; the results of PV values showed the DM played a dominant role. The breakdown viscosity (BD) and setback viscosity (SB) indicate the disintegration and association of starch during pasting; respectively. The BD and SB were also critical in determining the final viscosity (FV). The BD of the composite gels decreased significantly as the addition of CP-AEP; CP-CSP and CWW-WSP; suggested that the higher shear resistance and lower swelling power and hydration compared to the MS; that could be related with the amylose leaching which was prohibited by the pectin; especially CP-CSP (Bao Zhang et al., 2018). The SB was related to reassociation of amylose molecules and short-term retrogradation of the gel. The result of MS-AEP showed an increase indicating that retrogradation was promoted; which is consistent with the reports of Pan et al. (2024). However; lower SB values of MS-CSP and MS-WSP were found; suggested that the short-term retrogradation of starch was restrained. The opposite SB trends for MS-AEP versus MS-CSP and MS-WSP point to divergent retrogradation pathways. CP-AEP carries high methylesterification (DM 45.8%) yet sparse RG-I branching (Ara + Gal/Rha 2.28); its methyl-ester groups likely anchor hydrophobically to starch surfaces while the linear backbone bridges amylose chains through hydrogen bonding; accelerating recrystallization. In contrast; CP-CSP and CWW-WSP share low DM (<30%) together with extensive RG-I side chains features that favor electrostatic repulsion; steric blocking; and preferential water binding; collectively hindering amylose reassociation. This interpretation draws support from hydrogen bond energies in Table 3; in which MS-AEP registers stronger inter-chain bonding (9.81 kJ/mol) against weakened interaction in MS-CSP (9.12 kJ/mol). The final viscosity (FV) represents retrogradation ability and recrystallization tendency. The CP-AEP; CP-CSP and CWW-WSP decreased the FV; suggesting an decrease in the short-term retrogradation of MS. Pasting temperature (PT) involve losing crystallinity during the hearting process. The introduction of CP-AEP; CP-CSP and CWW-WSP slightly increased the PT from 91.10 °C to 91.85 °C; 92.65 °C and 94.25 °C; respectively. The results were similar with report; indicated the coverage and interaction of RG-I to starch improved the resistance to heating (Yin et al., 2021).

**Table 3:** The degree of short-range order, hydrogen bond energies and distances of composite gels.

Sample	R_1047/1022_	Inter-stand hydrogen bond			Inter-double helices hydrogen bond		
		Bond position (cm^−1^)	E (kJ/mol)	R (Å)	Band position (cm^− 1^)	E (kJ/mol)	D (Å)
MS	0.497 ± 0.034^c^	3515.0792	9.7032	2.8208	3282.2095	26.4507	2.7682
MS-AEP	0.653 ± 0.021^b^	3513.6267	9.8076	2.8205	3280.2376	26.5925	2.7678
MS-CSP	0.634 ± 0.045^b^	3523.2131	9.1182	2.8227	3289.9119	25.8968	2.7700
MS-WSP	0.785 ± 0.041^a^	3521.6637	9.2296	2.8223	3280.1761	26.5970	2.7678

### 3.3 Dynamic rheological properties

Dynamic rheological properties can reflect the three-dimensional network of composite gels (Ma et al., 2019). The G′ was an indicator of amylose aggregation in the short term retrogradation while the G′′ was used to evaluate the energy lost as reversible deformation appeared (Zhang et al., 2020).

**Fig. 3:**
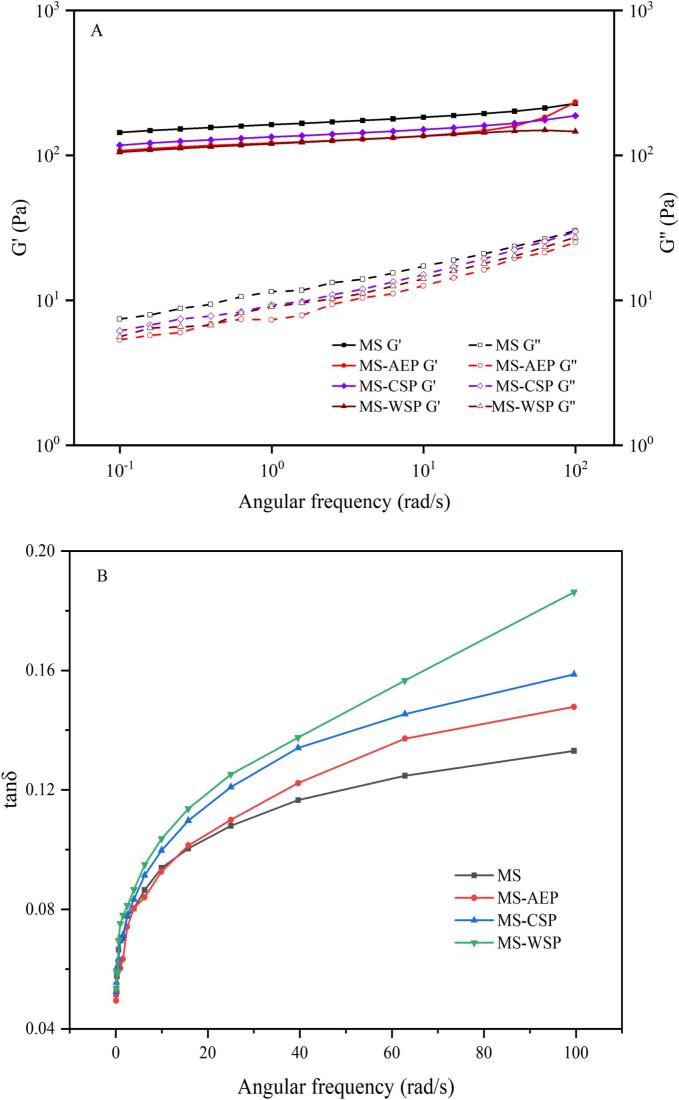
The storage modulus (G'), loss modulus (G") (A) and the loss angle tangent (tanδ) (B) of MS-pectin composite gels.

The angular frequency dependence of G′ and G′′ is shown in Fig.3A. With the increase in frequency, the G′ and G′′ of all gels increased, the G′ of all gels was higher than G′′ and no crossover phenomenon between G′ and G′′ appeared, illustrating that MS and MS-pectin composite gels exhibited solid-like behavior and belonged to a typical weak gel system (Kong et al., 2024). Compared with MS; the the G′ and G′′ of MS-AEP; MS-CSP and MS-WSP were significantly lower; Among them MS-AEP showed the lowest G′ and G′′; attributed to hydrophobic interference with amylose junction zones; MS-CSP exhibited intermediate values due to partial electrostatic compensation; MS-WSP displayed the highest tan δ; reflecting RG-I-mediated steric entanglement and viscous dissipation. These results indicate that DM-driven hydrophobicity and RG-I architecture dictate network disruption modes; rather than uniform water competition; the similar results were observed in low molecular weight pectin (DM < 50%) with wheat starch (Chen et al., 2023). Different from the commercial pectin; which increased the G′ of starch-pectin mixtures; the effect may be attributed to decrease in the number of starch linking regions caused by adsorption of pectin on the surface of MS and inhibition of amylose gelation. As shown in Fig.3B; values of tanδ were less than 1; indicating that the formation of elastic gel. Compare with MS; tan δ values of all MS-pectin mixtures increased; indicating that the gel structure was more viscous when pectin from CW participated in gelatinization. The tan δ values increased with the RG-I content; the entanglement between the chains of pectin and MS may be not conducive to the formation of a solid-like gel; since RG-I have more branched chains and showed anionic electricity; which means the interaction in strong and makes the gel structure viscous (Yin et al., 2021).

### 3.4 FT-IR of composite gels

#### 3.4.1 FT-IR spectrum

**Fig. 4:**
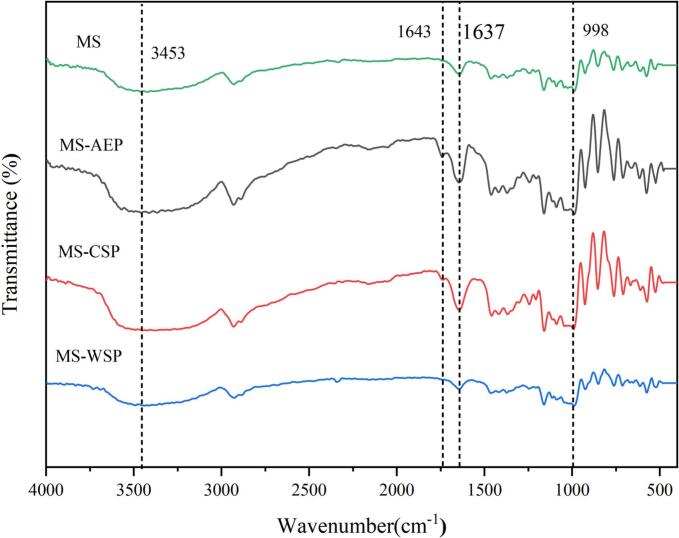
The FT-IR spectrum of MS and composite gels.

To investigate the interaction between MS and pectin from CW, the results of MS and composite gels are showed in Fig.4. In comparison to MS, a new peak at about 1743 cm^−1^ appeared in the FT-IR spectrum after adding the CP-AEP and CP-CSP, which was attributed to the ester carbonyl group of pectin. The intensity of MS-AEP was higher than MS-CSP, that might be attribute to the higher DM values. However, in the spectrum of MS-WSP, the absorption at 1743 cm^−1^ was much more smaller through the DM values was high than MS-CSP, the results suggested that the ester carbonyl group of CWW-WSP participate in the interaction with MS during gelatinization. Besides the characteristic peak at 1743 cm^−1^, no novel peaks appeared implied that pectin from CW interacted with MS via hydrogen bonding, electrostatic forces and hydrophobicity, which were all non-covalent bonds (Song et al., 2024). The peaks at 3452 cm^−1^ and 1643 cm^−1^ are attributed to intermolecular hydrogen bonds and bound water present in the gels respectively; while the peak intensity at 998 cm^−1^ is associated with intramolecular hydrogen bonding (Liu et al., 2025). The intensity of MS-CSP were strongest, showed that the hydrogen bonds formed between CP-CSP and starch molecules and the ester carbonyl group promoted the formation of hydrogen bonds. The gradual attenuation of the peak at 1643 cm^−1^ of MS-WSP indicated that CWW-WSP/starch hydrogen bonding competed with starch for water, resulted by the coverage effect on starch granule.

#### 3.4.2 Degree of short-range order

The peaks at 1047 cm^−1^ and 1022 cm^−1^ were attributed to the ordered and amorphous structure of starch, respectively (Liu et al., 2017). The FT-IR spectra were subjected to deconvolution and the ratios of bands at 1047 cm^−1^ and 1022 cm^−1^ were showed in Table 3. Compare to MS (0.497), the MS-AEP (0.653), MS-CSP (0.634) and MS-WSP (0.785) had a significantly increased in short-range order. It indicated that the addition of pectin improved the interactions between amylose and amylopectin to form ordered crystalline structure through hindering the starch-water interaction. As reflected by pasting properties (FV and BT) and the bound water, MS-WSP showed the strongest hydration and encapsulation effect, that may be the reason for highest amount of short-range order of MS-WSP. Also, the the amylose leaching amount and complex index between starch and pectin were responsible for the short-range ordered structure. It was reported that the higher DM could bonded with leached amylose by hydrogen thus promote the short-range order formation, while the lower DM exhibited the opposite effect (Chen et al., 2023). Due to the anionic electric and branched chemical structure of RG-I, the interaction of CWW-WSP and starch molecules promoted the molecular folding, formation of helical structure and the rearrangement of the disrupted crystal.

#### 3.4.3 Hydrogen bonding energy and distance

**Fig. 5:**
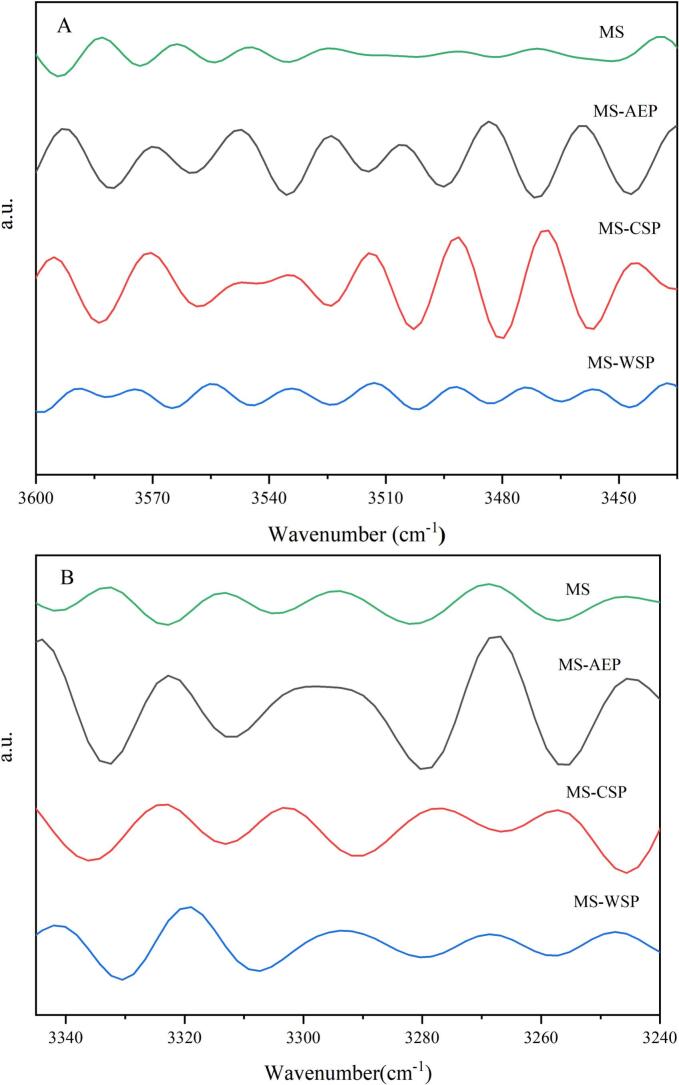
The second derivative of the FI-IR spectrum at different ranges of composite gels. A (3600 cm^−1^-3450 cm^−1^), B (3350 cm^−1^-3520 cm^−1^).

The inter-stand hydrogen bonds and inter-double helices hydrogen bonds were corresponded to the 3515 cm^−1^ and 3286 cm^−1^ at the second derivative spectra of FT-IR spectra, respectively (Hao, Rongrong, Ranran, & Yaoqi, 2021). The positions of hydrogen bonding, energy and distance were calculated and showed in Fig.5 and Table 3. The shift of bands at 3515.1 cm^−1^ and 3282.2 cm^−1^ to 3513.6 cm^−1^ and 3280.2 cm^−1^ was found in MS-AEP, indicating that hydrogen bands of inter-strand and inter-double helices were enhanced, which meant the aggregation of helices structure, reduced the distance and high energy between hydrogen bonds (Chen et al., 2023). The results may be ascribed to the DM values, the carbonyl participated in the formation of hydrogen bonds endowed great bond energy. Both the CP-CSP and CWW-WSP made the bonds of hydrogen move to higher bonds, indicated a decrease and looseness of hydrogen bonds in composite gels, while inter-double helices hydrogen bonds of MS-WSP showed opposite results, suggesting enhanced the doubles helices hydrogen bonds. The results may be attributed to the high water-binding ability and hydrophobic interaction of RG-I (Zhang et al., 2023). The interchain hydrogen bond energy of MS-AEP is 9.8076 kJ/mol, representing a 1.08% increase compared to MS (9.7032 kJ/mol). whereas the interchain hydrogen bond energy of MS-CSP is 9.1182 kJ/mol, representing a 6.0% decrease relative to MS. This variation directly reflects the regulatory effect of DM values on hydrogen bonding: CP-AEP with high DM enhances interchain hydrogen bonds in starch chains, whereas CP-CSP with low DM attenuates this hydrogen bonding interaction.

### 3.5 Microstructure of pectin from CW and composite gels

**Fig. 6:**
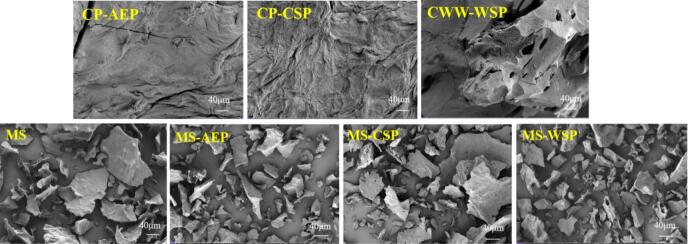
Scanning electron microscopy observations of pectin from CS, MS and composite gels.

The microstructure of pectin and freeze-dried composite gels at the resolution of 40 μm was showed in Fig.6. The CP-AEP and the CP-CSP presented obvious sheet structure with some bumps and kept relatively intact, the CP-CPS showed some filamentous structure closed against the surface. However, the CWW-WSP appeared holed block-like structure. This results showed that the surface morphology of pectin with different extract method were different. As for the composite gels, all samples showed broken pieces, the MS-AEP, MS-CSP and MS-WSP resulted in smaller pieces than MS. Also, the MS-AEP showed obvious network structure with “honeycomb” shape, the MS-CSP presented obvious surface coating with irregular shape. It was reported that the LM of pectin could result in incomplete gelatinization due to strong water absorption (Yang et al., 2022), the rough surface of MS-CSP indicated the tight coverage of pectin on the starch gels. However, the MS-WSP showed penetration into the starch gels, the “honeycomb” shape was also observed. The different type of interaction also consistent with the results of pasting properties.

### 3.6 XRD of MS and composite gels

**Fig. 7:**
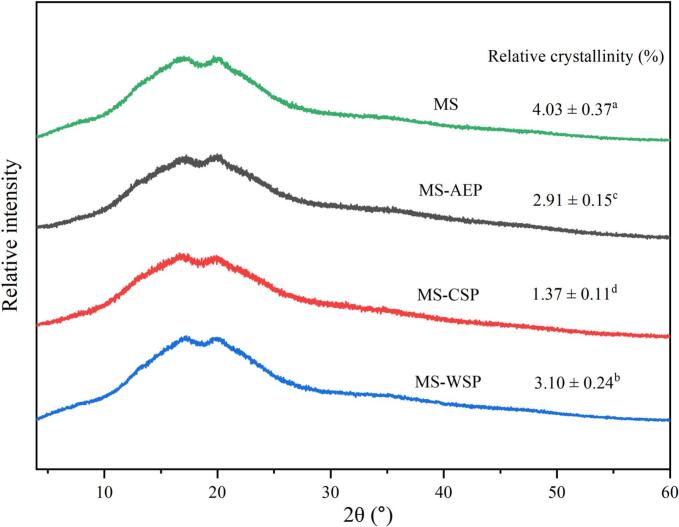
X-ray diffractograms and relatively crystallinity of MS and composite gels.

The X-ray diffractograms and relative crystallinity of MS and composite gels was showed in Fig.7. The peak position showed the MS gels was a typical B-type starch with peaks at 2θ close to 17°and 20°, which is a double helix structure formed by retrograded amylose and amylopectin (Du et al., 2019). The unchanged diffraction peak position suggested that addition of CP-AEP; CP-CSP and CWW-WSP did not transfer the crystal type of MS. Compared with MS (4.03%); the relative crystallinity of MS-AEP; MS-CSP; and MS-WSP decreased to 2.91%; 1.37%; and 3.10% respectively. This was because the LM pectin was more prone to adsorb onto the surface of MS rather than form hydrogen bonds with MS (Chi et al., 2021). However; the MS-WSP exhibited higher relative crystallinity than MS-AEP and MS-CSP. The results indicated that hydroxyl group of RG-I interacted with amylose and retarded the reassociation of starch chains (Yang et al., 2025).

### 3.7 ^13^C CP/MAS spectra of MS and composite gels

**Fig. 8:**
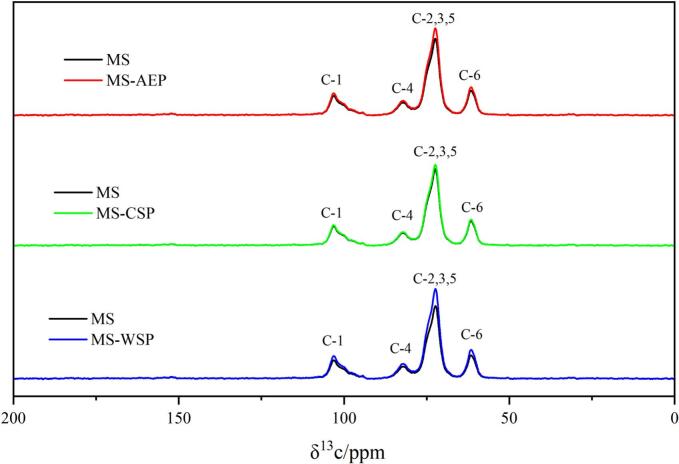
^13^C CP/MAS spectra of MS and composite gels.

The molecular structures of starch and composite gels were determined by the ^13^C CP/MAS. The spectra were showed in Fig.8, peaks at 103.15, 81.96, 72.30 and 61.45 ppm were attribute to C-1, C-4, C2,3,5 and C-6 of MS (*A.* Flores-Morales et al., 2012). A signal intensity of all peaks in MS-WSP and MS-AEP was observed; while the MS-CSP showed almost the same intensity. The position of C-1 peaks suggested the addition of CP-AEP; CP-CSP and CWW-WSP did not change the crystal type of MS gel; which was confirmed by the XRD results. Also; the increase of intensity in C-1 showed the CP-AEP and CWW-WSP play an positive role in the molecular packing of helices in the crystalline regions; which was consistent with results of the inter-double helices hydrogen bond (Jinglin et al., 2009).The peak at 81.96 ppm was characteristic only for the amorphous starches; the results indicated that the CP-AEP; CP-CSP and CWW-WSP had no obvious effects on the amorphous state of starch (Flores-Morales, Jimenez-Estrada and Mora-Escobedo, 2012). The strong and sharp peak of C2;3;5 is a typical B-type characteristic (Atichokudomchai et al., 2004), the differences observed in ^13^C CP/MAS NMR spectra suggested that the CP-AEP and CWW-WSP promoted the double helix content formation rather than the relative crystallinity. The peak intensity of glucose C-6 from CP-AEP and CWW-WSP was stronger might be attributed to the -CH_3_ groups due to DM and RG-I, respectively.

## 4 Conclusions

This study investigated the pectin from CW and the impact of pectin on MS gels. The three pectin extracts decreased the viscosity of composite gels, the addition of pectin inhibit the leaching of amylose and the network formation due to the low-ester values and result in competition for water available. The CP-AEP could maintain the viscoelasticity of composite gels better than CP-CSP and CWW-WSP. Compared with MS, MS-AEP, MS-CSP and MS-WSP decreased the relative crystallinity but increased the degree of short-range order. The CP-AEP enhanced the inter-stand hydrogen bond and inter-double helices hydrogen bond, CWW-WSP weakened the inter-stand hydrogen and enhanced the inter-double helices hydrogen bond, indicating that both DM and RG-I palyed an important role in interaction with MS. The MS-AEP, MS-CSP and MS-WSP maintained the B type characteristic, and CP-AEP and CWW-WSP promote the formation of helices hydrogen bond in crystalline regions but decreased the relative crystallinity. These structure-dependent interactions offer targeted strategies for modulating starch-based food textures using coffee waste pectins.

## CRediT authorship contribution statement

**Wei Zhang:** Writing – original draft. **Chunyan Zhang:** Data curation. **Jiahe Dai:** Investigation. **Danxia Shi:** Writing – review & editing. **Fang Yang:** Software. **Hong Li:** Supervision. **Xiaohui Liu:** Resources, Conceptualization.

## Declaration of competing interest

The authors declare that they have no known competing financial interests or personal relationships that could have appeared to influence the work reported in this paper.

## Data Availability

Data will be made available on request.
