# Supplementary material for: Extraction and characterization of pectin from coffee waste and the effects on pectin-maize starch gel
Source: Food Chem X. 2026 Mar 30;35:103804. doi: 10.1016/j.fochx.2026.103804 (PMC13081701; doi:10.1016/j.fochx.2026.103804)
Supplement: Supplementary file 1 — Supplementary material [file mmc1.docx]

**
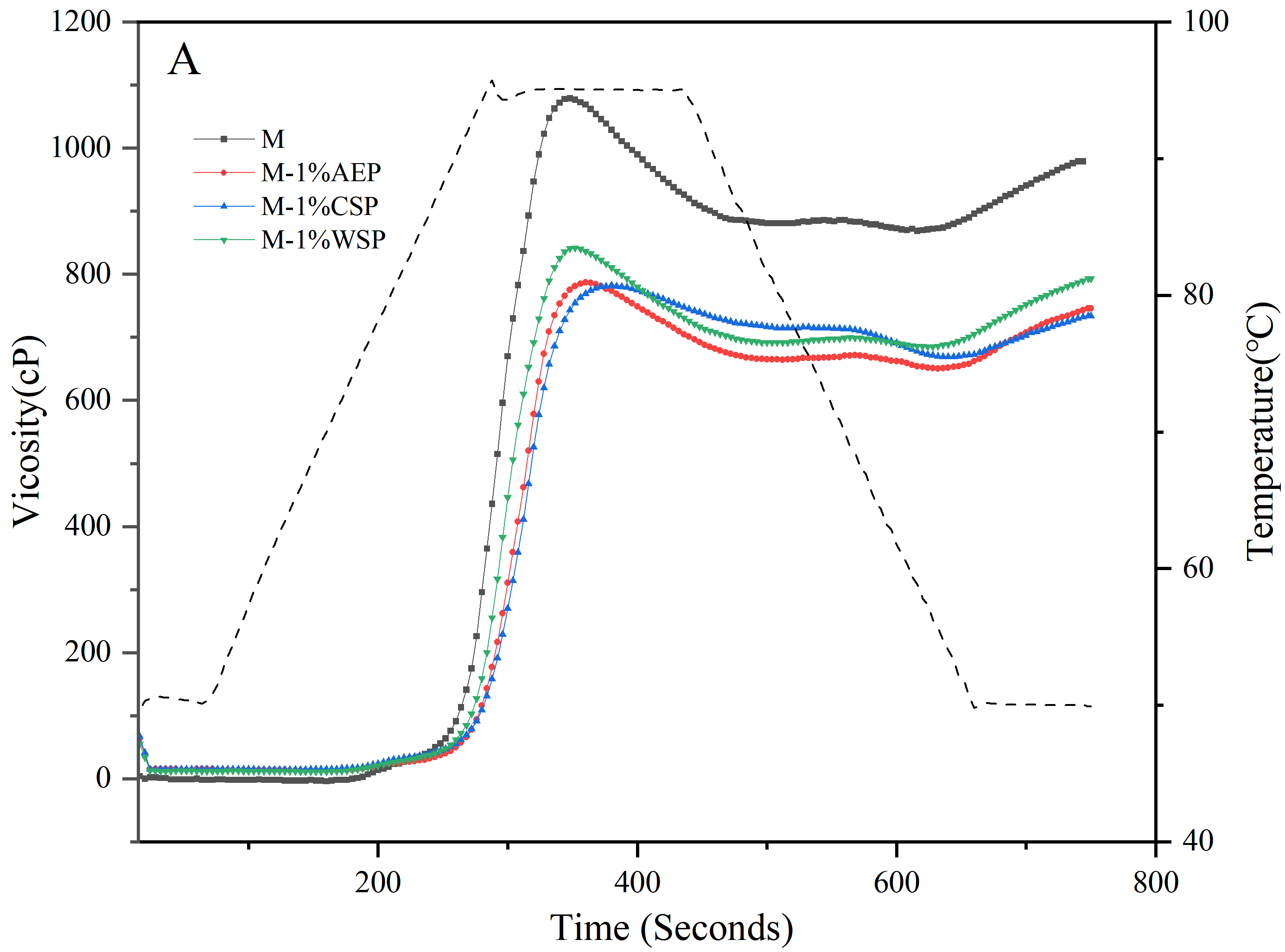
**

**
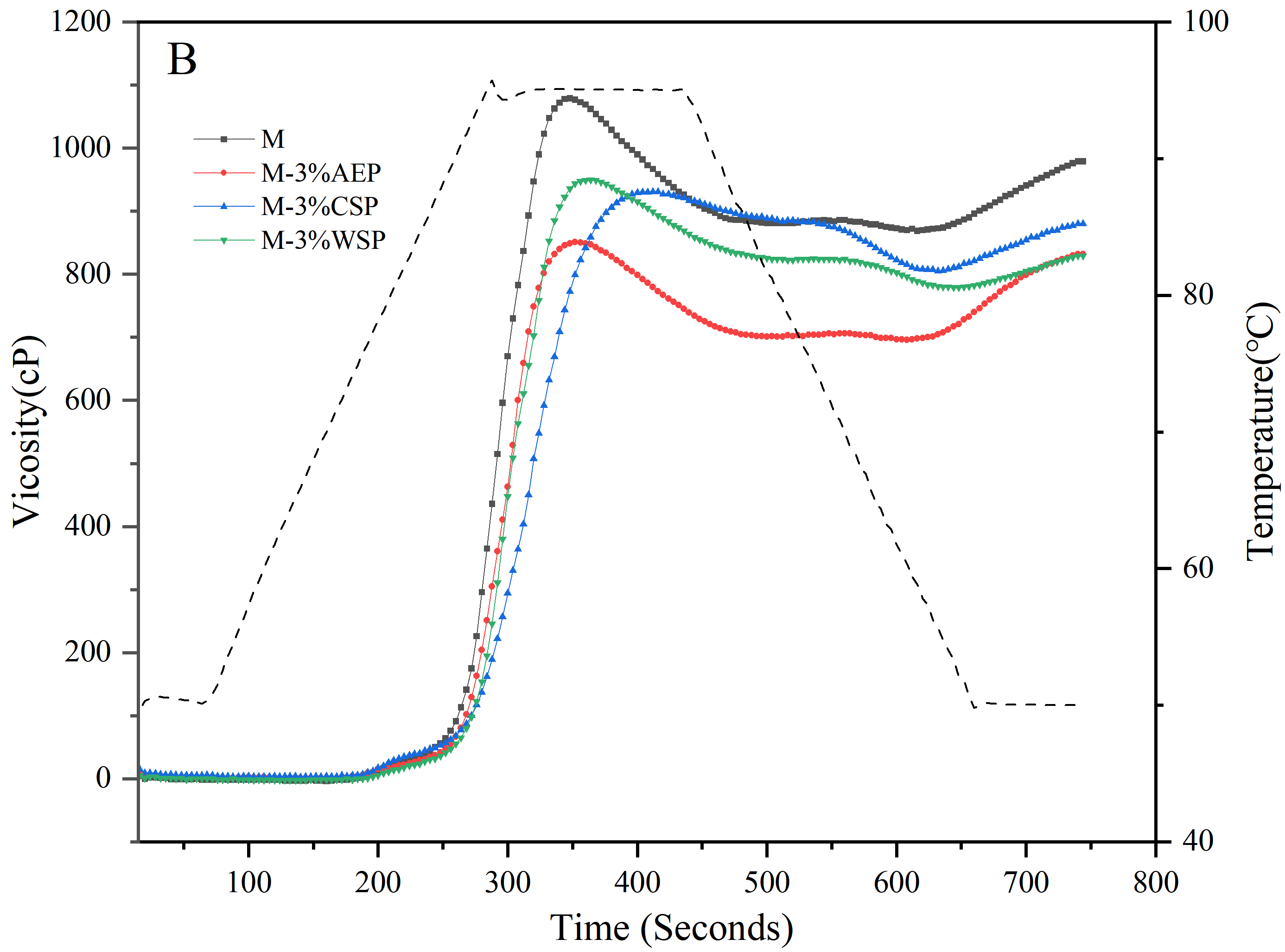
**

**
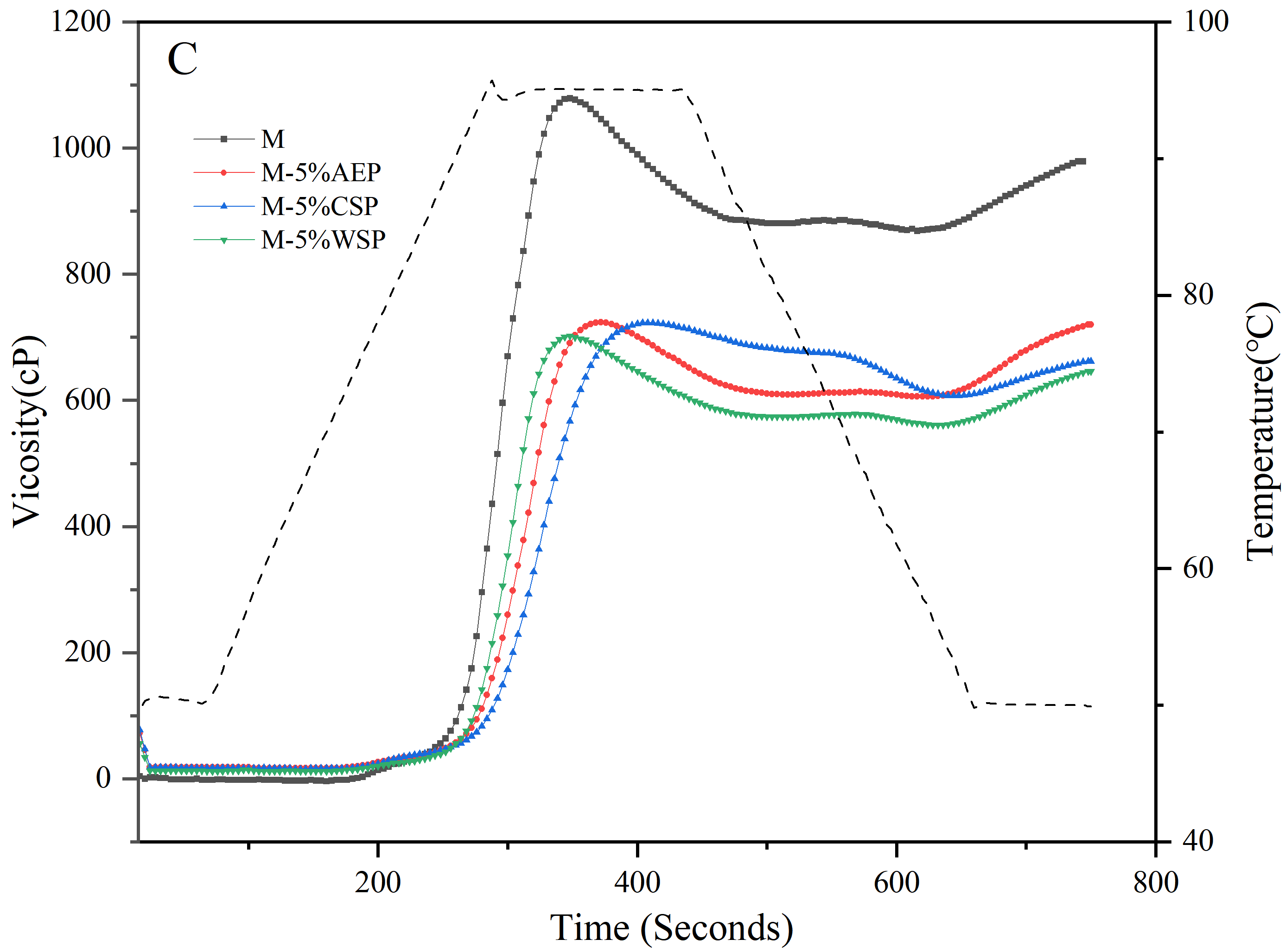
**

**Fig.S1.** Pasting curves of maize starch (MS) and MS-pectin mixtures with different pectin concentrations 1%, 3%, and 5%


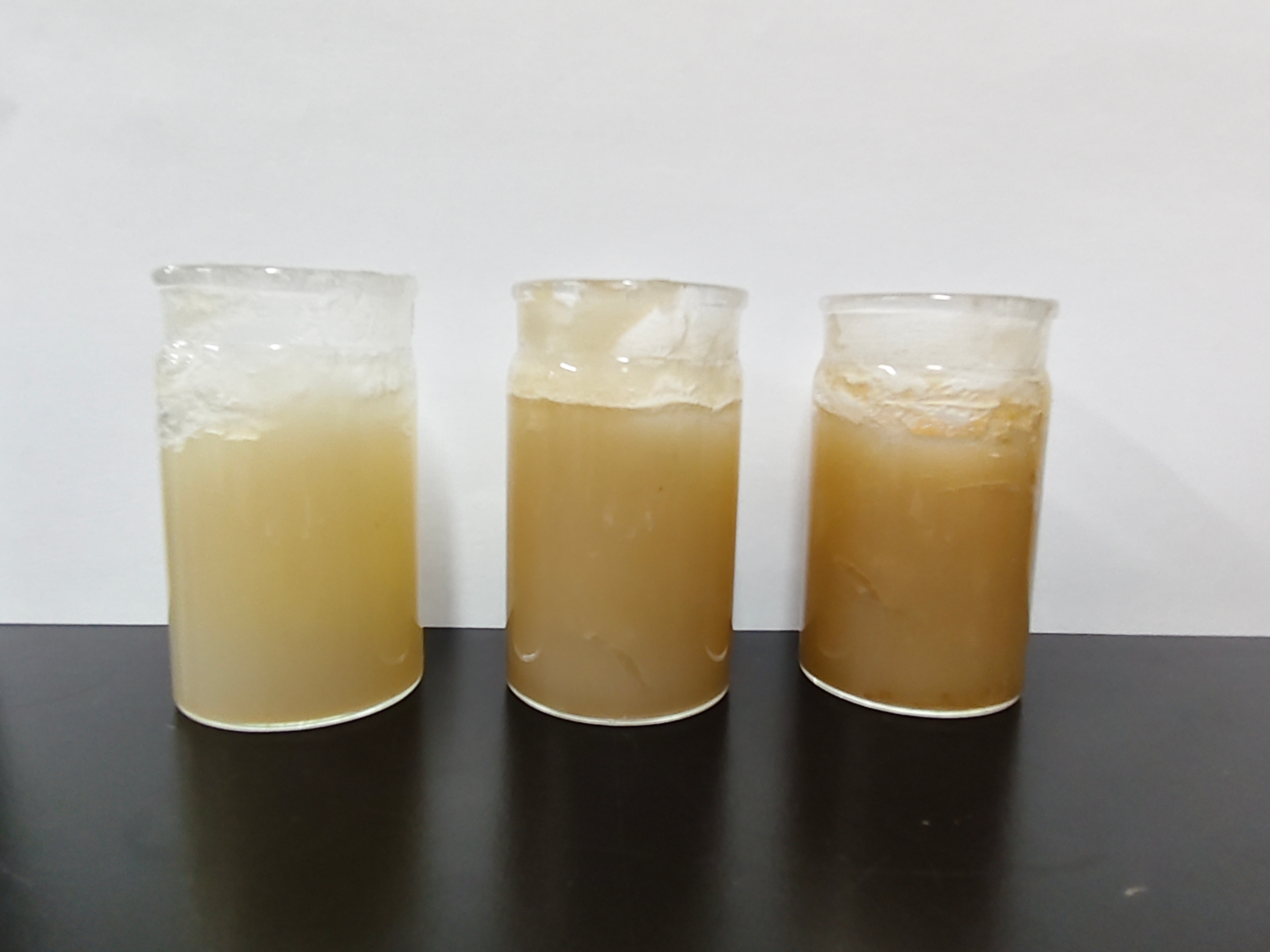


MS-AEP

1%

3%

5%


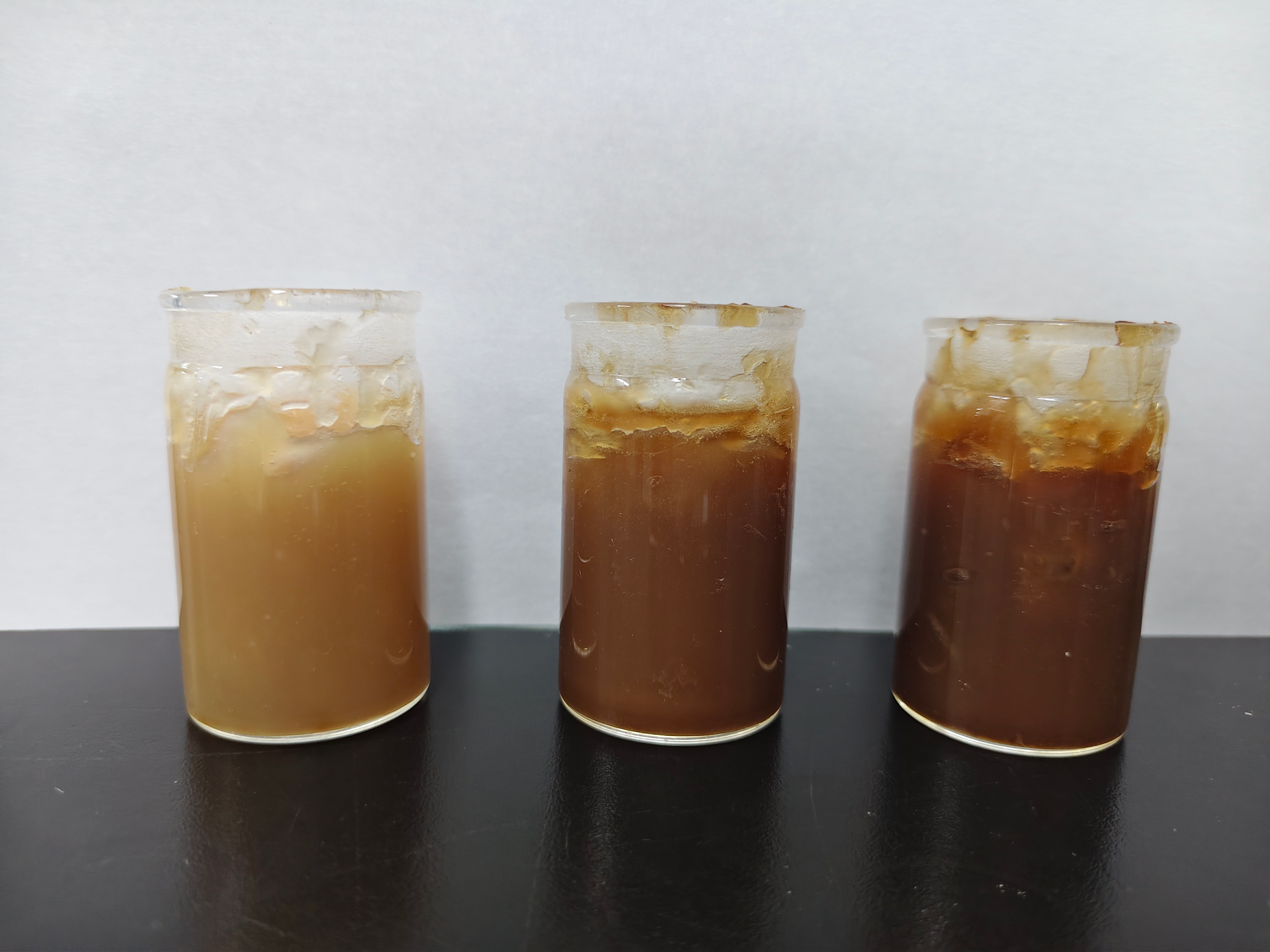


MS-CSP

1%

3%

5%


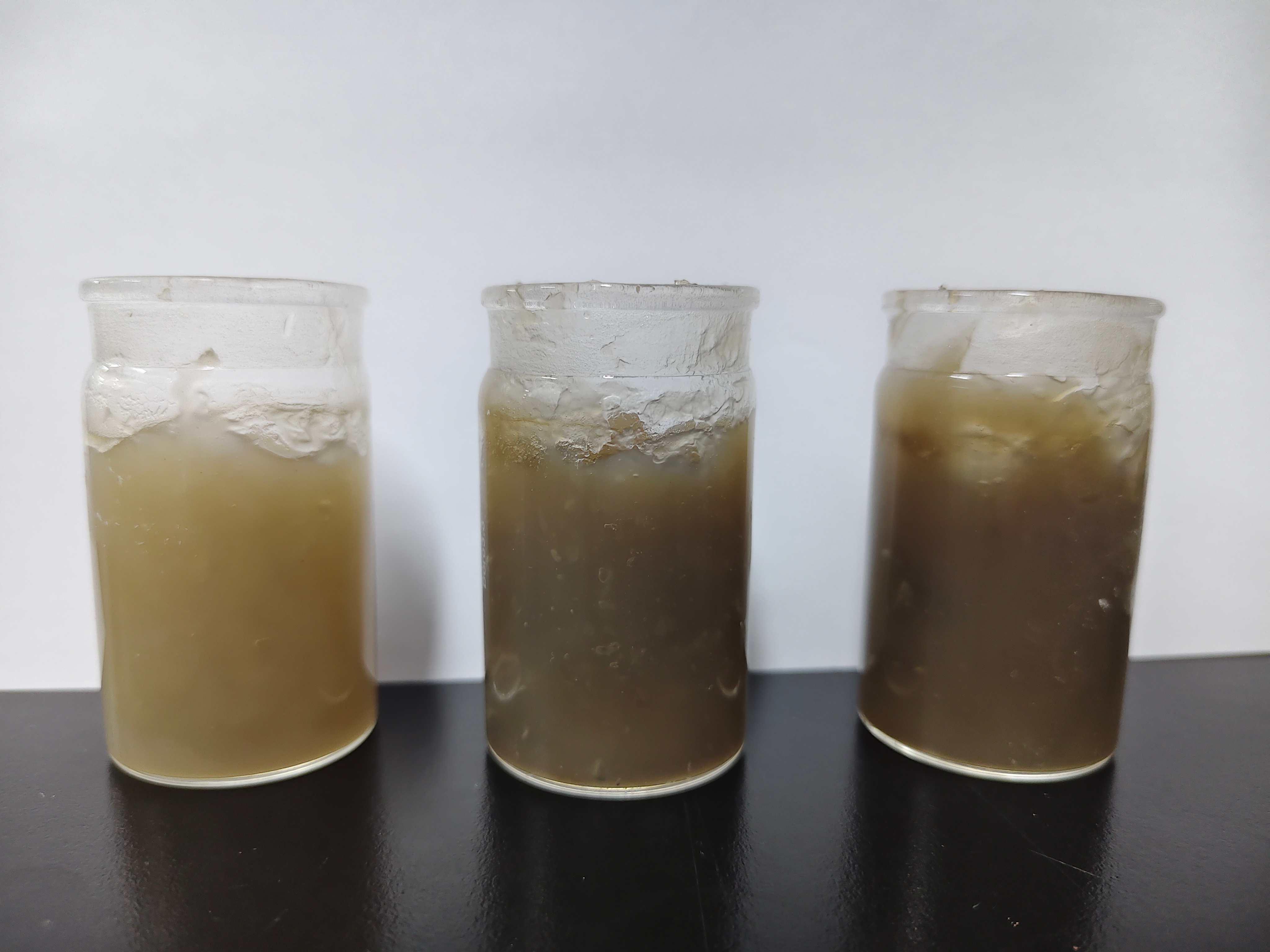


MS-WSP

1%

3%

5%

**Fig.S2.** The photo of maize starch (MS) and MS-pectin mixtures with different pectin concentrations 1%, 3%, and 5%
